# Post In-Hospital Cardiac Arrest Simulation to Improve Medical Decision Making and Communication in Internal Medicine Residents

**DOI:** 10.15766/mep_2374-8265.11642

**Published:** 2026-10-16

**Authors:** Emily Harris, Alexandra Collis, David Carlbom, Nicholas Johnson, James Town, Vincent Raikhel

**Affiliations:** 1 Fellow, Division of Pulmonary, Critical Care and Sleep Medicine, University of Washington; 2 Clinical Assistant Professor, Department of Medicine, University of Washington; 3 Associate Professor, Division of Pulmonary, Critical Care and Sleep Medicine, University of Washington; 4 Professor, Department of Emergency Medicine, Division of Pulmonary, Critical Care and Sleep Medicine, University of Washington; 5 Associate Professor, Division of Pulmonary, Critical Care and Sleep Medicine, University of Washington; 6 Assistant Professor, Department of Medicine, University of Washington

**Keywords:** Simulation, Cardiac Arrest, Critical Care, Internal Medicine, Clinical/Procedural Skills Training

## Abstract

**Introduction:**

Cardiac arrest is a common cause of morbidity and mortality in hospitalized patients, and is a critical event that hospital-based physicians must be prepared to manage. While cardiac arrest care follows established algorithms, management of patients after return of spontaneous circulation (ROSC) is more dynamic and nuanced. Resident physicians therefore need structured training to provide high-quality post-ROSC care.

**Methods:**

We developed a 90-minute simulation session on postarrest care for internal medicine interns. The session included 2 simulation cases: one involving a patient with ventricular tachycardia arrest in the intensive care unit, and another featuring a patient with pulseless electrical activity arrest in an acute care setting. Interns alternated between roles as participants and observers to both practice and analyze communication and clinical skills. Between simulation cases, a brief teaching talk was delivered to review a framework for post-ROSC care. The curriculum was evaluated using pre- and postsession questionnaires to evaluate changes in knowledge and confidence.

**Results:**

Thirty-four learners participated in our curriculum over 2 academic years. We observed a statistically significant increase in the percentage of learners who correctly answered knowledge-based questions about target blood pressure (50% to 100%) and oxygen saturation (22% to 74%) in the post-ROSC period. We also saw significant increases in learners’ self-reported confidence in communication (28% to 74%) and ability to generate a differential diagnosis for a cardiac arrest (25% to 74%).

**Discussion:**

Our simulation session increased intern knowledge and confidence in managing patients in the post-ROSC setting.

## Educational Objectives

By the end of this activity, learners will be able to:

1. Demonstrate giving and receiving handoff for a post–return of spontaneous circulation (ROSC) patient to/from another medical team, including relevant events leading up to the cardiac arrest, diagnostic results, therapeutics initiated, and communicate differential diagnosis for the cardiac arrest.
2. Formulate a differential diagnosis and evaluate for reversible causes of cardiac arrest in the immediate post-ROSC period.
3. Choose initial therapeutics needed to stabilize hemodynamics and arrhythmias in an unstable patient immediately after resuscitation from cardiac arrest.

## Introduction

Cardiac arrest is a common cause of morbidity and mortality, affecting more than 350,000 people outside of the hospital and an estimated 200,000 hospitalized patients annually in the United States.^1,2^ Survival to discharge after in-hospital cardiac arrest (IHCA) ranges between institutions, but is typically estimated to be close to 20%.^3^ A number of variables, including patient comorbidities, initial cardiac rhythm, and etiology of arrest, have been associated with outcomes.^2^ Research has also shown that the existence of designated code blue response teams is associated with improved survival after IHCA.^4^ Given the tenuous clinical status of patients who have suffered IHCA, timely and organized care in the immediate post–return of spontaneous circulation (ROSC) period is critical. Data have demonstrated that hypotension, hypoventilation, and hyperventilation in the immediate post-ROSC period are associated with worse outcomes.^5^ Further, targeted temperature management—which requires strict control of patient body temperature – has been shown to improve neurologic outcomes in the post-ROSC period.^6^

While care for patients during cardiac arrest is guided by standardized algorithms, the care of patients who achieve ROSC is less formulaic, although the American Heart Association (AHA) and the Neurocritical Care Society (NCS) have created specific guidelines for management in the post-ROSC period.^7-9^ More recently, post–cardiac arrest syndrome has been recognized as a clinical entity with specific recommendations and evidence-based management strategies.^10,11^ These include supporting hemodynamics with both fluid resuscitation and vasopressors, maintaining normal oxygenation and ventilation, and temperature control to support neurologic recovery. Post-ROSC clinical care is typically carried out by an intensive care unit (ICU) team; however, the care of patients during a cardiac arrest and in the immediate post-ROSC period often involves multiple handoffs and collaboration between cardiac arrest response (code blue), ICU, and acute care medicine teams. Practices and preferences around handoff vary between contexts and individuals. Carr et al.^12^ and Raikhel et al.^13^ found that physicians caring for patients in the emergency department after out-of-hospital cardiac arrest prioritized initial rhythm, post-ROSC ECG, and witness-reported events at the time of arrest as key pieces of information. While checklists have been proposed as helpful tools for facilitating communication of important information between teams in the post-ROSC period,^14^ implementing best practices for communication and handoff during acute hospital-based emergencies is a critical skill that requires deliberate training and practice to ensure patient safety.

At most academic institutions, resident physicians serve as members of designated code blue teams. However, given their frequent clinical rotation, they may have limited experience and low levels of comfort with resuscitation and post-ROSC care. As a result, the providers responsible for cardiac arrest resuscitation and postarrest care are often relative novices, and without dedicated training during residency many physicians will enter the workforce as independent practitioners who must learn to manage these tenuous clinical scenarios on the job. High-fidelity simulation is a well-established tool for preparing trainees for such high-risk, low-frequency patient care events by providing a realistic and dynamic environment in which to practice without compromising patient care.^15-17^ Simulation has been incorporated into an increasing number of GME curricula and specialties for these reasons.^18^ While simulations for code blue scenarios and other critical clinical events have been published, we identified only 1 published simulation curriculum for care of patients in the immediate post-ROSC period.^19-23^ To fill this critical gap and prepare resident physicians to manage patients in the post-ROSC period, we created a post-ROSC simulation session that aims to provide a framework for the initial care of patients after achieving ROSC.

## Methods

### Development

The post-ROSC simulation session curriculum was created by hospitalists, pulmonary and critical care medicine (PCCM) physicians, and emergency medicine and critical care physicians who are all faculty members at the University of Washington (2 hospitalists, 2 PCCM faculty, 1 emergency medicine and critical care faculty, 1 PCCM fellow). The 2 hospitalists co-direct a longitudinal simulation-based curriculum for internal medicine interns that focuses on a variety of hospital-based clinical emergencies, and that increases in complexity throughout the course of the academic year. One PCCM physician is the medical director of the medical intensive care unit (MICU) at 1 of the training hospitals where internal medicine residents routinely care for patients in the MICU. The emergency medicine and critical care physician and other PCCM faculty member have both served as the chair of the code blue committee at 1 of the training hospitals.

The simulation was developed in 2024 and piloted at 1 of our training hospitals. The simulation session was refined and implemented in 2025 at all 3 hospitals, which serve as training sites for the University of Washington internal medicine residency program, all of which are tertiary care centers. They consist of an academic tertiary care medical center, a public safety net hospital, and a Veterans Affairs hospital affiliated with our university. Interns in the internal medicine residency at our institution rotate through all 3 of these hospitals during their PGY-1 year. Each session lasted approximately 90 minutes and was attended by 5-12 learners.

Learners during this session consisted of interns rotating on inpatient admitting services and inpatient consulting services at the 3 hospitals within the University of Washington training program. Interns were primarily in the internal medicine residency program, but other residency programs (anesthesia, psychiatry, neurology, physical medicine and rehabilitation, and family medicine) were also included if they were rotating on general medicine ward services at the time of implementation. Participation was required as part of the internal medicine residency intern curriculum, and senior team members (senior residents and attendings) covered patient care responsibilities to facilitate intern attendance.

This simulation session was held near the end of the academic year, with the primary goal of increasing intern confidence with leading code response teams and caring for patients after ROSC is achieved. The session taught a framework for clinical management of patients after ROSC and included opportunities to develop the communication and team-based skills required for effective management of cardiac arrest and post-ROSC scenarios. One of the hospitals in our system had previously adopted a post-ROSC checklist of critical tasks, and we used the items on this checklist as part of the framework for this session.^14^ This session was held separately from advanced cardiac life support training sessions that occur for all residents at the end of their PGY-1 year.

### Equipment/Environment

The simulations were all performed in dedicated simulation labs designed to mirror resources of an acute care medical/surgical ward. They included:

• Simulated environment: telemetry floor
• Equipment:
  ○ High-fidelity human patient simulator
  ○ Defibrillator with adult pads
  ○ Monitor for real-time simulated monitoring of the heart rate, blood pressure, oxygen saturation, and temperature
  ○ Telemetry leads
  ○ Medication syringes and IV bags
  ○ Liter bags of fluid
  ○ IV tubing and IV poles
  ○ Nasal cannula tubing
  ○ Hospital gown
• Standardized participants:
  ○ One standardized nurse (a facilitator) who administered all medications, IV fluids, and blood products, and provided imaging, lab results, and limited medical history
  ○ One standardized consultant (a facilitator) who acted as a critical care fellow, cardiology fellow, or anesthesiologist depending on which teams the participants chose to consult during the case

### Personnel

Two faculty members were present as facilitators for each session. The faculty included hospitalists and 1 PCCM fellow. The high-fidelity human patient simulator was run with the help of simulation technicians. Two embedded participants played by the facilitators acted as a bedside nurse and consulting fellow.

### Implementation

The simulation session was structured to include a brief introduction before proceeding with 2 simulation cases, each of which was followed by a debrief with an intervening teaching talk reviewing a post-ROSC framework (Appendix A). Our introduction began with an overview of the simulation facility and equipment. Within the introduction, we highlighted the fiction contract and importance of psychological safety and active participation.^24^ Learners were then divided into 2 groups of 3-6 individuals. One group was selected as the active participants for the first simulation case. A volunteer was elicited from the group to serve as the team leader for the simulation. Active participants who were not serving as the leader were instructed to act as internal medicine interns who responded to a code blue page to help the leader, but they were not assigned a specific role. We intentionally did not assign alternative role titles (eg, nurse, respiratory therapy, critical care fellow) due to past observations that learners were distracted by the specific role responsibilities. One of the 2 facilitators served as a bedside nurse during the simulation. The other served as the consultant who answered consult calls during the simulation and acted as a critical care fellow who entered the room and asked for a patient summary at the completion of the simulation. The learners who were not active participants in the first simulation case were able to observe the simulation from a separate room with audio and video connection to the simulation lab.

The first case began with the team positioned outside the room and being called inside by the standardized nurse. Learners were told that a patient had just had a brief cardiac arrest and ROSC had been achieved. The case was presented in the simulation case file, which was used by the facilitators (Appendix B). Participants were given the patient's sign out documentation immediately before entering the simulation room (Appendix C). Supplemental data for each case, including ECGs, chest radiographs, and laboratory results, was available to learners if requested (Appendix D).

Following the first simulation case, the facilitators led a structured debrief with both groups, guiding discussion of teamwork, communication, and clinical care during the simulation (Appendix E). This debrief included a review of the critical action checklist for the case (Appendix F). After the debrief, a separate teaching “chalk talk” to introduce a framework for post-ROSC care was given to the group by the facilitators (Appendices G and H).

After the debrief and chalk talk, the second simulation case proceeded in the same manner as the first case (Appendices I-K). During the second simulation case, the group who were the initial observers became the active participants, and they were observed by the group who had actively participated in the first case. A second debrief followed the second simulation case (see Appendix E).

### Debriefing

After each simulation, a faculty facilitator led a structured debrief (see Appendix E). The debrief was divided into 2 parts. The first part included teamwork and communication skills. The second part included clinical reasoning, medical knowledge, and application. During the second part of the debrief, the critical action checklists for each case were reviewed (Appendix L, see Appendix F). Our structured debrief was based on PEARLS (Promoting Excellence and Reflective Learning in Simulation).^25^ Each part of the debrief began with a discussion of what had gone well and was followed by discussion of areas for improvement. The perspectives of the team leader, active participants, and observers were elicited by the facilitator.

### Assessment

Interns were given voluntary anonymous pre- and postparticipation surveys to complete (Appendix M). The preparticipation survey was given immediately prior to the session, and the postparticipation survey was given immediately after the debrief of the second simulation case. Participants were provided with a QR code link to the surveys to be completed on their smartphones. Participants were still allowed to participate in the simulation if they declined to complete the surveys. Learners who arrived late to the activity were not given preparticipation surveys to avoid disrupting the flow of the simulation activity, and their postparticipation survey data was excluded from our analysis. Surveys covered learners’ confidence in managing post-ROSC care for a patient, communicating with other providers, and generating a differential for etiologies of a cardiac arrest. Each question was answered on a 5-point Likert scale (1 = *not at all*, 2 = *not very*, 3 = *neutral*, 4 = *somewhat*, 5 = *very*). These responses were grouped into “not confident” (1-3) and “confident” (4-5). Pre- and postsimulation responses were then compared using a chi-square analysis to determine whether there was a statistically significant increase. Because of the relatively small number of participants surveyed, we did not analyze the degree of change between presimulation and postsimulation confidence and knowledge.

We also asked participants 3 multiple-choice knowledge-based questions regarding hemodynamic and respiratory targets in post-ROSC management. These specific parameters were chosen based on the internal post-ROSC checklist used at our institution, and the number of knowledge-based questions was limited to preserve time for the simulation activity.^14^ Responses were grouped into correct and incorrect responses, and the pre- and postsimulation responses were analyzed using chi-square analysis to determine whether there was a statistically significant change.

Lastly, we solicited open-ended comments about what learners gained from the session and how it will change their practice.

## Results

Thirty-four learners participated in the simulation session during the 2024 and 2025 academic years. Thirty-two learners (94%) completed the pre- and postparticipation surveys.

We observed a statistically significant increase in correct responses to all 3 multiple-choice knowledge-based questions, which included the recommended partial pressure of carbon dioxide in arterial blood (PaCO2), mean arterial pressure (MAP), and peripheral oxygen saturation (SpO2) for patients in the post-ROSC setting (Table, see Appendix M). Ninety-four percent of learners (30 of 32) reported that the session improved their confidence in caring for patients in the post-ROSC period. A majority of interns reported that they thought the session improved their communication skills (27 of 32, 84%) and leadership skills (24 of 32, 75%). When learners were asked to rate their confidence in a variety of management situations, there was a statistically significant increase in learners’ reported confidence in managing a patient after a pulseless electrical activity (PEA) arrest (pre: 5 of 32, post: 23 of 32, *P* < .01) and ventricular tachycardia (VT)/ventricular fibrillation (VF) arrest cardiac arrest (pre: 3 of 32, post: 21 of 32, *P* < .01). We also observed a statistically significant increase in confidence generating a differential diagnosis for cardiac arrest (pre: 8 of 32, post: 27 of 32, *P* < .01) and providing handoff to another medical provider on a patient who had just obtained ROSC (pre: 9 of 32, post: 27 of 32, *P* < .01). Lastly, there was a significant increase in learners who felt confident using a defibrillator (pre: 12 of 32, post: 26 of 32, *P* < .01). Twenty-nine of 32 learners (91%) said that they would like to attend more simulation sessions similar to this one.

**Table.**
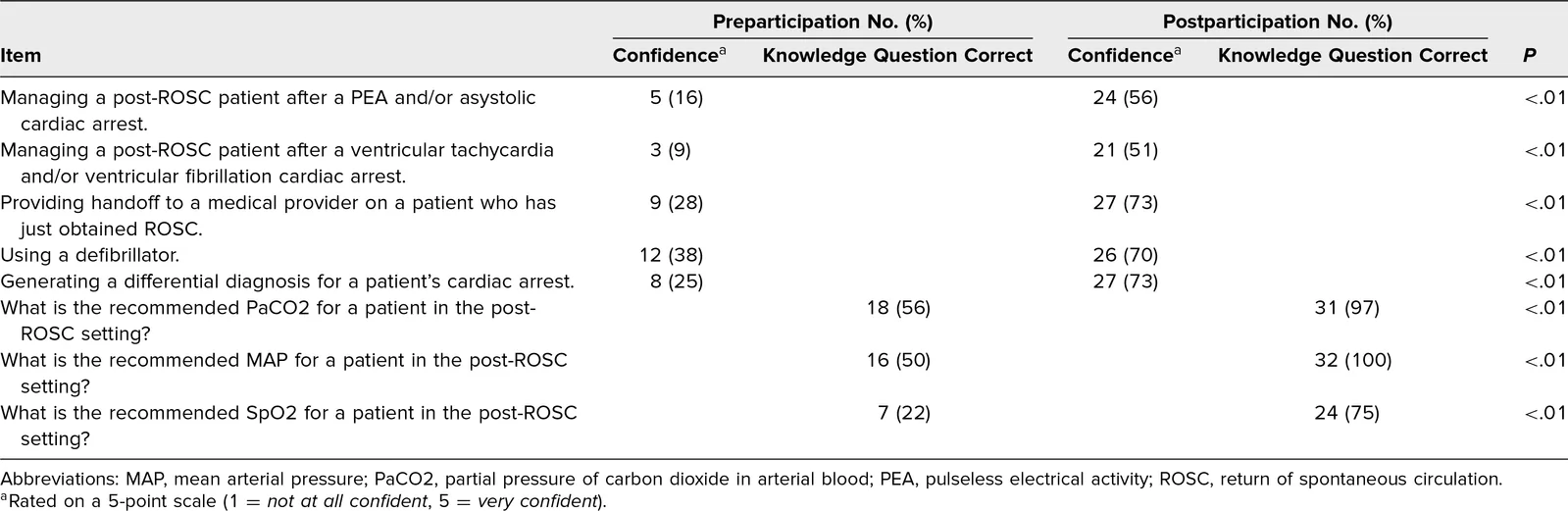
Survey Results

Representative responses included “[A] framework for thinking about post PEA arrest” and comments about when to deliver synchronized cardioversion versus unsynchronized defibrillation to patients. When asked for open-ended feedback about the session, representative comments included positive feedback about the organization of the session: “All instructors were really welcoming and educative [educational]. It helped doing one case without instruction then debriefing/chalk talk and then doing another case to practice what we learned.” We also received positive feedback regarding the simulation environment: “Really comfortable and welcoming environment to learn and make mistakes to be able to grow and do a sim in real time.” There were also comments expressing a desire for more simulation sessions during the academic year.

## Discussion

Caring for patients who have experienced IHCA and achieve ROSC is a low-frequency event in the acute care hospital setting, but it is critical for patient survival and therefore a vital skill for inpatient acute care providers. Structured, guideline-directed care in the immediate post-ROSC period is crucial and can prevent recurrent cardiac arrest and improve patient outcomes.^26,27^ It is known that resident physicians feel ill-prepared for the task of leading code blue teams during their training despite receiving training on this topic.^28^ Our presimulation survey data demonstrated that our residents reported a lack of confidence in many key components of post-ROSC care.^29^ Despite the importance of post-ROSC care, little has been done to address the knowledge and confidence gap of trainees in this area.

Intern knowledge of basic care goals in the post-ROSC setting and confidence in managing a patient post-ROSC improved following our simulation curriculum. While simulation curricula for leading cardiac arrest responses exist,^20-22^ our simulation is unique in that it focuses on care of patients after ROSC is achieved and provides a framework for caring for patients who are post-ROSC. Our curriculum provides 2 simulation sessions with a didactic chalk talk between the 2 sessions, allowing for spaced repetition of the material. The variety of teaching modalities also accommodates a variety of learning styles.

The structure of our curriculum allowed learners to observe simulation cases even if they were not directly participating. The ability to observe the actions of their peers and the results of those actions allowed for vicarious reinforcement, a key tenet of social learning theory. Additionally, this allowed us to include a greater number of learners in each simulation session, addressing the limitations on resident time that many programs face. Debriefing each simulation case and discussing the actions, communication skills, and clinical outcomes learners experienced and observed during the case allowed for the consolidation of information from the observation and modeling. The debriefs after each simulation case also allowed for learners to receive feedback on their performance and learn from observing peers.

Our chalk talk is designed to provide learners with a straightforward cognitive framework for managing patients in the post-ROSC period, including evaluating for reversible/treatable causes of cardiac arrest, and goal targets for hemodynamic and ventilator management. We also included a communication element to remind learners to communicate with the appropriate parties (eg, the primary clinical team, the patient's family, and other teams). Following the didactic chalk talk, learners were given the opportunity to apply the framework in a second simulation case, which encouraged deliberate practice.^30^ The spaced repetition of simulation cases and interleaving of teaching modalities may have led to greater retention of material for learners.

Learners in our curriculum were placed in simulated clinical scenarios that they were unlikely to have experienced prior, particularly in leadership roles. Understandably, it was not expected that learners would effectively and independently complete tasks without assistance. Timing this session toward the end of the PGY-1 year was an intentional effort to allow this session to engage learners in their zone of proximal development.^31^ Learners were encouraged to look to each other for guidance, as well as engage and utilize other resources to navigate these challenging cases. Our didactic chalk talk also provided guidance in the form of a cognitive framework for post-ROSC care. In the postsimulation survey, learners reported greater confidence in clinical management of patients in the post-ROSC period. Prior work has demonstrated that inexperienced learners who participate in simulation curricula that force them to operate in the zone of proximal development find the experiences to be valuable, instructional, and motivating. Residents in our innovation likely experienced similar benefit, which is reflected in the postsimulation survey data.^32^

Our postsimulation debrief conversations included reflection on the emotional experiences of learners in addition to the cognitive processes used in each case. Because our cases simulated a high-stakes clinical scenario that learners were unlikely to have experienced prior, they provoked stress and anxiety in learners. However, residents also reported that they found the curriculum to foster a safe environment in which to practice fundamental skills. In our experience, the practice of beginning each simulation session by reviewing the fiction contract and establishing the session as a safe space by normalizing mistakes and asking participants to avoid discussion of the session afterward has been foundational in fostering a safe learning environment.^24^ It is possible that discussion of the emotional and cognitive experience contributed to increased self-efficacy and increased confidence.

Our innovation has several limitations. First, while residents reported significantly increased confidence in several specific domains of post-ROSC patient care, confidence does not necessarily reflect skill, and it is not clear whether increases in learner-reported confidence will be associated with improvements in patient care. We evaluated objective measures of retention by including multiple-choice knowledge questions in our survey and also saw a significant increase in correct answers to all of these questions, though notably our knowledge assessment was limited to specific qualifiable parameters of post-ROSC care. Our curriculum was implemented in 1 residency program at 1 institution, and this may limit the generalizability of our findings to different specialties or at different institutions. In particular, we recognize that the resources available at our tertiary care center, such as a cath lab and a range of consulting specialties, are not universal in all practice environments. Planning, organizing, and implementing high-fidelity simulation sessions is also a resource-intensive endeavor that requires facilitator expertise, and this may not be readily available at all programs. Lastly, while our simulation and curriculum reflect current AHA and NCS guidelines for post-ROSC care, these guidelines are periodically updated. To ensure that the simulation session remains accurate and relevant, we recommend referencing the most recent AHA and NCS guidelines and updating the simulation cases and post-ROSC framework to reflect any future clinical updates.

Caring for patients in the immediate post-ROSC period is of critical importance to patient survival following cardiac arrest and a fundamental skill for a variety of hospital-based medical specialties. Further investigation is needed to evaluate the impact of the curriculum on resident behavior, patient care outcomes, and whether our intervention is effective for learner cohorts in other educational environments.

## Appendices


Session Flow Guide.docxCase 1 Facilitator Guide.docxCase 1 Patient Sign Out.docxCase 1 Supplemental Data.docxDebrief Guide.docxCase 1 Critical Action Checklist.docxPost-ROSC Care Framework.docxPost-ROSC Care Framework Facilitator Guide.docxCase 2 Facilitator Guide.docxCase 2 Patient Sign Out.docxCase 2 Supplemental Data.docxCase 2 Critical Action Checklist.docxPre- and Postsimulation Survey.docx
*All appendices are peer reviewed as integral parts of the Original Publication.*
