# Supplementary material for: Post In-Hospital Cardiac Arrest Simulation to Improve Medical Decision Making and Communication in Internal Medicine Residents
Source: MedEdPORTAL. 2026 Oct 16;22:11642. doi: 10.15766/mep_2374-8265.11642 (PMC13674318; doi:10.15766/mep_2374-8265.11642)
Supplement: Supplementary file 1 [file mep_2374-8265.11642-s001.zip › A. Session Flow Guide.docx]

**Appendix A: Post ROSC Session Flow**

| **Session section** | **Duration** | **Facilitators needed** | **Appendix needed** |
| --- | --- | --- | --- |
| Introduction | 10 minutes | 1 | none |
| Simulation 1 | 15 minutes | 3 facilitators: mannequin technician, standardized RN, standardized consultant | B, C, D, F |
| Debrief 1 | 15 minutes | 1 Facilitator to run the debrief | E |
| Post-ROSC care framework | 15 minutes | 1 Facilitator to give the chalk talk | G, H |
| Simulation 2 | 15 minutes | 3 facilitators: mannequin technician, standardized RN, standardized consultant | I, J, K, L |
| Debrief 2 | 15 minutes | 1 Facilitator to run the debrief | E |
