# Supplementary material for: Post In-Hospital Cardiac Arrest Simulation to Improve Medical Decision Making and Communication in Internal Medicine Residents
Source: MedEdPORTAL. 2026 Oct 16;22:11642. doi: 10.15766/mep_2374-8265.11642 (PMC13674318; doi:10.15766/mep_2374-8265.11642)
Supplement: Supplementary file 1 [file mep_2374-8265.11642-s001.zip › B. Case 1 Facilitator Guide.docx]

**Appendix B: Case 1 Facilitator Guide**

| **Simulation case title: Sepsis Strikes Back** | |
| --- | --- |
| **Patient name: Ian Aurora**  **Patient Age: 84** | |
| **Brief narrative description of case** | Ian Auroa is an 84-year-old man with a past medical history of type 2 diabetes mellitus, chronic obstructive lung disease, atrial fibrillation, heart failure with reduced ejection fraction (EF of 35%) status post implantable cardioverter- defibrillator placement, and gastroparesis admitted for concern for a gout flair of the right ankle. 30 minutes ago, a rapid response was called altered mental status. No interventions were initiated during the rapid response and the AMS was thought to be due to delirium. After the rapid response team left, the patient became hypotensive and had a pulseless electrical activity cardiac arrest. They received two rounds of CPR before return of spontaneous circulation was obtained. |
| **Learning Objectives** | - Demonstrate obtaining a broad diagnostic work up in the immediate post ROSC period to determine any reversible causes of a cardiac arrest. - Implement initial therapeutics needed to stabilize a patient’s hemodynamics who has just obtained the return of spontaneous circulation. - Deliver an organized and succinct hand off for a post ROSC patient that includes relevant events leading up to the cardiac arrest, key diagnostic results, therapeutics initiated, and differential diagnosis for the cardiac arrest. |
| **Critical Actions** | - Identify self upon entering the room and assign roles. - Obtain hand off from medical team members. - Perform focused physical exam and obtain data from existing monitors. - Obtain a broad diagnostic work up to determine the etiology of the cardiac arrest. - Articulate a differential diagnosis for PEA cardiac arrest that includes sepsis. - Treat shock with IV fluids and vasopressors. - Start broad spectrum antibiotics - Provide sign out to MICU provider |
| **Page to Code Blue Team** | “Code Blue, pt Auroa” |
| **Patient Medications** | Allopurinol 100 mg daily  Apixaban 5mg BID  Empagliflozin 10 mg  Insulin aspart 5 units TID AC  Insulin glargine 9 units QHS  Ipratropium bromide/albuterol q6h PRN  Metoprolol Succinate 50 mg daily  Prednisone 30 mg daily  Sacubitril / Valsartan 24/26 mg BID  Tamsulosin 0.4 mg daily |
| **Patient AM Labs** | WBC 5.3 K/uL, Hgb 13 g/dL, Hct 41%, Plts 315 K/uL    Na 132 mEq/L, K 4.0 mEq/L, Cl 97 mEq/L,  HCO3 15 mEq/L, BUN 32 mg/dL, Cr 1.1 mg/dL,  Glucose 314 mg/dL |
| **Learner Preparation** | None |
| **Manikin setup on learner arrival** | - Monitor: patient is attached to monitor with pulse, blood pressure, oxygenation, and temperature displayed. - The patient is in bed at 0 degrees. - A chest compression backboard should be under the patient and defibrator pads are on the patient. - Blood pressure cuff is applied to patient. - Pulse oximeter is attached to patient/continuous O2 monitoring is set up - Pulse: patient has a regular tachycardic pulse with rate in ~90s - Access: one 20g PIV present, one in right AC - Exam: eyes are half open - Standardized STAT RN is feeling the left carotid pulse. |
| **Standardized Participants** | - STAT RN: provides verbal cues to learners if needed - MICU fellow: obtains sign out from learners at the end of the simulation. - Consultants: available by phone (can be the same person who has the role of the MICU fellow) |
| **Materials Needed** | • Manikin  • Blood pressure cuff  • IV Fluids  • Antibiotics   - Vasopressors - Defibrillator with pads - Copy of ACLS post arrest care algorithm |
| **Timing of medication arrival.** | • IV Fluids should be provided to the medical team immediately upon request.  • Antibiotics and vasopressors should be provided 1-2 minutes after request.   - Any other requested medications should be administered 1-2 minutes after request. |
| **Timing of new data.** | • Laboratory data should be provided to the medical team 1 minute after requested.  • ECG should be provided at the time it is requested.  • CXR should be provided 1 minute after requested.  • Any requested culture data does not result during simulation.   - Ultrasound is out of service and cannot be utilized during this simulation. |

| **Physical Examination** | |
| --- | --- |
| **INITIAL VITAL SIGNS** | Temperate 39.1; Heart Rate 110 BP 70s/50s; MAP<65; RR 12; SpO2 96% on 4L NC |
| **General** | Drowsy, lying in hospital bed, eyes half open |
| **Lungs** | normal respiratory rate, rhonchi heard on right side, no wheezes |
| **Cardiovascular** | Tachycardic, regular rhythm, no extra heart sounds |
| **Abdomen** | Flat, non-distended, normal bowel sounds |
| **Neurological** | Moans to tactile stimulation but not to verbal stimulation, no focal deficits on limited exam, pupils react to light appropriately |
| **Extremities** | No edema, extremities are warm to the touch, right ankle is swollen and red. Patient moans if the right ankle is manipulated. |
| **Skin** | Warm, erythema surrounding the right ankle |
| **Psychiatric** | Minimally response |

| **Case Stage/ Time Point** | **Case Details** | **Additional Information** |
| --- | --- | --- |
| **State 1: Learner enters room and begins care of patient.** | - T 39.1; HR 110s BP 70s/50s; MAP<65; RR 12; SpO2 96% on 4L NC - When learners enter the room STAT RN reports “the patient was altered and then started to become hypotensive, they had a PEA arrest and we got ROSC right before you arrived.” - The patient has a pulse throughout state 1. This can be confirmed by either the STAT RN or bedside RN. - If learners ask for the MICU or anesthesia the learners should be given the phone number to call the standardized consultant. | **Trigger Points:**   - If learners administer fluids (30 ml/kg) OR norepinephrine blood pressure increases to 80s/60s for 1 minute before returning to 70s/50s. - If learners start IV fluids AND norepinephrine proceed to state 2. - If the tasks needed to proceed to state 2 are not completed within 8 minutes, proceed to state 3. |
| **State 2: Improving circulatory shock** | - T 39.2; HR 120s BP 110s/70s; RR >12; SpO2 97% - The patient has a pulse throughout state 2. | **Trigger Points:**   - After 10 minutes proceed to state 4   **OR proceed to state 4 earlier if the leaner has completed the following tasks:**   - Discussed a differential diagnosis for cardiac arrest that includes sepsis - Obtained relevant diagnostic data. - Delegated a team member to update the patients surrogate decision maker. - Initiated broad spectrum antibiotics. |
| **State 3: Patient goes into atrial fibrillation with rapid ventricular response and deteriorating hemodynamics.** | - Vital signs: T 38.8 C BP 60s/40s, HR 130-140s, O2 saturation 60s%, on 4L NC. - Patient goes into atrial fibrillation with a rapid ventricular rate. - The patient has a pulse throughout state 3. - If the patients perform a synchronized cardioversion that patient will return to sinus tachycardia with a heart rate in the 110s. Blood pressure improves to 80s/50s. - The patient has a pulse throughout state 3. | **Trigger Points:**   - After 10 minutes proceed to state 4   **OR proceed to state 4 earlier if perform the following actions:**   - Synchronized cardioversion of the patient. - Start IV fluids (30 mL/kg), AND norepinephrine. |
| **State 4: Hand off to the MICU fellow** | - A standardized MICU fellow enters the room and asks for a summary of the situation. - If not discussed, MICU fellow should ask learners for events of the cardiac arrest, diagnostics obtained, therapies started, and differential diagnosis. |  |
| **Case Ends** |  |  |

**Ideal Scenario Flow***.*

The learners enter the room to find a patient in shock after a PEA cardiac arrest. They immediately recognize that the patient is hypotensive and initiate hemodynamic support by starting a bolus of IV fluids and norepinephrine. The learners verbalize a differential diagnosis that systemically goes through the Hs and Ts as possible causes of the patient's cardiac arrest. They complete a physical exam and obtain diagnostic data that increases the concern for sepsis. Given the patients' hemodynamic instability and unresponsive mental status, learners consult anesthesia for consideration of intubation and explain their rationale for thinking the patient needs to be intubated. The learners consult the MICU for transfer and provide complete sign out to the critical care fellow to facilitate transfer of patient to the MICU.

**Anticipated Management Mistakes**

1. Learner’s perform unsynchronized cardioversion while patient has a pulse: Learners have been confused about the indications for synchronized vs unsynchronized cardioversion. Despite the patient having a pulse in this case, learners have performed an unsynchronized cardioversion. We have had the patient go into ventricular fibrillation without a pulse when this occurs.
2. Failure to consult the necessary teams. Some simulation participants have not consulted anesthesia for intubation, or the MICU for transfer. If this occurs, the standardized STAT RN can prompt the participants to consult these teams verbally suggesting it after 7 minutes within the simulation.
3. Medical management of an unstable tachyarrhythmia. Learners have attempted to medically manage with patient in state 3 where the patient is presenting with an unstable tachyarrhythmia. The patient's hemodynamics continues to deteriorate if this form of management is attempted. If the learners want to administer a second medication after the first has not worked, the standardized STAT RN can suggest that the patient should be cardioverted.

**MICU fellow consult:** If the learners call for a MICU consult, they should be provided a phone number to be able to reach the standardized MICU fellow who should be in a different room than the simulation. Upon picking up the phone the standardized MICU fellow should obtain information regarding key patient past medical history, events leading up to the cardiac arrest, details of the cardiac arrest, as well as diagnostic data obtained after ROSC was achieved. These details should be requested if they are not offered by the learner making the call. Learners should be asked to provide their differential diagnosis for the patient. After this information is obtained, the standardized MICU fellow should tell the learner they will be at bedside shortly.

**Anesthesia consultant:** If the learners call for anesthesia, they should be provided with a phone number to be able to reach anesthesia who should be in a different room than the simulation. Upon picking up the phone the standardized anesthesiologist should obtain information regarding key patient past medical history, events leading up to the cardiac arrest, details of the cardiac arrest, as well as diagnostic data obtained after ROSC was achieved. Learners should provide their thoughts on whether intubation is required now and the reasons why. After this information is obtained, the standardized anesthesiologist should tell the learner they will be at bedside shortly.

**Case 1 supplemental data** should be provided to simulation participants if requested. Laboratory diagnostic data should be cut apart prior to the simulation so that the specific laboratory studies requested by the participants can be provided. Diagnostic studies that are not requested by the participants should not be provided. All laboratory, electrocardiogram, and radiograph diagnostic data should be provided to the participants 1-2 minutes after being requested.

| **Point-of-Care Glucose** | 294 mg/dL |
| --- | --- |
| **Complete Blood Count** | WBC 23.94 K/uL, Hgb 12.3 g/dL,Hct 42%, Plts 512 K/uL |
| **Basic Metabolic Panel** | Na 124 mEq/dL, K 5.1 mEq/dL, Cl 92 mg/dL, HCO3 10 mEq/L, BUN 31 mg/dL, Cr 3.2 mg/dL, Ca 8.7mg/dL, Mg 1.9 mg/dL |
| **B-type Natriuretic Peptide** | 35 pg/mL |
| **Troponin-I** | 41.8 ng/L |
| **Liver Function Tests** | AST 131 U/L, ALT 192 U/L, AlkPhos 132/ TBili 2.4 |
| **Coagulation Studies** | APTT 34.8 secs, PT 11.9 secs, INR 1.1 |
| **Arterial Blood Gas** | pH 7.12, pCO2 31 mmHg, pO2 127mmHg , HCO3 11 mEq/L |
| **Lactate** | 6.3 mmol/L |

**Post Cardiac Arrest Chest X-Ray**

Image by Kirollos Bechay, retrieved from: https://radiopaedia.org/cases/pacer-pads-and-automated-implantable-cardioverter-defibrillator-in-intubated-patient?lang=us on 12/1/25. Licensed under https://creativecommons.org/licenses/by-nc-sa/3.0/.

**Chest radiograph from before hospitalization**

Image by Jeremy Jones, retrieved from: https://radiopaedia.org/cases/dual-lead-cardiac-pacemaker on 12/15/25. Licensed under https://creativecommons.org/licenses/by-nc-sa/3.0/.

**Post ROSC ECG, Stages 1 and 2**

**Image**

**Case courtesy of life in the fast lane:** <https://litfl.com/atrial-fibrillation-ecg-library/> accessed on 12/15/25. Licensed under https://creativecommons.org/licenses/by-nc-sa/4.0/.

**Post ROSC ECG Stage 3**

**Provide this ECG to participants if the case has proceeded to stage 3.**

**Case courtesy of life in the fast lane:** <https://litfl.com/atrial-fibrillation-ecg-library/> accessed on 12/15/25. Licensed under https://creativecommons.org/licenses/by-nc-sa/4.0/.

**Admission ECG**

**Provide this electrocardiogram to participants if they request an electrocardiogram from the hospital admission.**

**Case courtesy of life in the fast lane:** <https://litfl.com/atrial-fibrillation-ecg-library/> accessed on 12/15/25. Licensed under https://creativecommons.org/licenses/by-nc-sa/4.0/.

**Case 1 patient sign out data.** This document should be provided to participants at the beginning of the simulation.

| **FLOOR – 6W** | **VITALS-LABS** | **Ins/Outs – Cultures** | **MEDS** | **COMMENTS/TASKS** |
| --- | --- | --- | --- | --- |
| Auroa, Ian  MRN01010101  DOB: 10/30/40, 84 M  Room: 6212-2  **Code: Full Code**  **Allergy: NKDA**  **Hospital Day 2**  Prob/Proc   - COPD - DMT2 - A fib - HFrEF - Gout | T 37.8 p 98 NIBP 113/72 r16 sat 99% on RA weight 234 lb  WBC 5.3 K/uL, Hgb 13 g/dL, Hct 41%, Plts315 K/uL    Na 132 mEq/L, K 4.0 mEq/L, Cl 97 mEq/L,  HCO3 15 mEq/L, BUN 32 mg/dL, Cr 1.1 mg/dL,  Glucose 314 mg/dL |  | Allopurinol 100 mg daily  Apixaban 5mgBID  Empagliflozin 10 mg daily  Insulin aspart 5 units TID AC  Insulin glargine 9 units QHS  Ipratropium bromide/albuterol q6h PRN  Metoprolol Succinate 50 mgdaily  Prednisone 30 mgdaily  Sacubitril / Valsartan 24/26 mg BID  Tamsulosin 0.4 mg daily | STABLE/ FULL  84-y/o M with a PMH of DMT2, COPD, afibon AC, HFrEF (EF of 35%) s/p ICD placement, and gastroparesis admitted for concern for a gout flair in teright knee  Today:  -pain improved, conton prednisone  -some confusion this afternoon, likely delirium  XC: NTD  If/then:  -if goes into RVR give 5 mg of metop |

| **Critical Action Checklist should be used by facilitators while observingthe simulation and used to support the debrief discussion after the simulation is completed. The content of the checklist should not be shared with simulation participants before the simulation.** | | | | | | |
| --- | --- | --- | --- | --- | --- | --- |
| **Post ROSC Case 1 Critical Action Checklist** | | | | **Date:**  **Site:**  **Leader:**  **Others:** | | |
| **Evaluator:** | | | |  |  |  |
| **Code Blue Team Leadership** | | | |  |  |  |
| 1. Identifies self as leader of code blue team | | | | | |  |
| 2. Asks for updated vital signs | | | | | |  |
| 3. Stands at foot of bed | | | | | |  |
| 4. Assigns roles (or identifies roles) | | | | | |  |
| 5. Asks for events leading up to code team arrival | | | | | |  |
| 6. Asks for current medication list | | | | | |  |
| 7. Asks for past medical history | | | | | |  |
| 8. Asks for recent labs | | | | | |  |
| **Initial post ROSC assessment** | | | | | | |
| 1. Verbalizes differential diagnosis going through Hs and Ts | | | | | |  |
| 2. Obtains electrocardiogram | | | | | |  |
| 3. Obtains chest radiograph | | | | | |  |
| 4. Assesses neurological status (examines pupils, mental status, gross motor function) | | | | | |  |
| **Labs** | | | | | | |
| Basic metabolic panel |  | Complete blood count |  | | Blood gas with lactate |  |
| **Treatment/drugs** | | | | | | |
| 4. IV fluids | | | | | |  |
| 5. IV pressors such as norepinephrine | | | | | |  |
| 6. Broad spectrum antibiotics such as cefepime +metronidazole, piperacillin and tazobactam, or carbapenem +/- vancomycin | | | | | |  |
| **Other Comments:** | | | | | | |
