# Supplementary material for: Post In-Hospital Cardiac Arrest Simulation to Improve Medical Decision Making and Communication in Internal Medicine Residents
Source: MedEdPORTAL. 2026 Oct 16;22:11642. doi: 10.15766/mep_2374-8265.11642 (PMC13674318; doi:10.15766/mep_2374-8265.11642)
Supplement: Supplementary file 1 [file mep_2374-8265.11642-s001.zip › C. Case 1 Patient Sign Out.docx]

**Appendix C: Case 1 Patient Sign Out Data**

This document should be provided to participants at the beginning of the simulation.

| **FLOOR – 6W** | **VITALS-LABS** | **Ins/Outs – Cultures** | **MEDS** | **COMMENTS/TASKS** |
| --- | --- | --- | --- | --- |
| Auroa, Ian  MRN01010101  DOB: 10/30/40, 84 M  Room: 6212-2  **Code: Full Code**  **Allergy: NKDA**  **Hospital Day 2**  Prob/Proc   - COPD - DMT2 - A fib - HFrEF - Gout | T 37.8 p 98 NIBP 113/72 r16 sat 99% on RA weight 234 lb  WBC 5.3 K/uL, Hgb 13 g/dL, Hct 41%, Plts 315 K/uL    Na 132 mEq/L, K 4.0 mEq/L, Cl 97 mEq/L,  HCO3 15 mEq/L, BUN 32 mg/dL, Cr 1.1 mg/dL,  Glucose 314 mg/dL |  | Allopurinol 100 mg daily  Apixaban 5mg BID  Empagliflozin 10 mg daily  Insulin aspart 5 units TID AC  Insulin glargine 9 units QHS  Ipratropium bromide/albuterol q6h PRN  Metoprolol Succinate 50 mg daily  Prednisone 30 mg daily  Sacubitril / Valsartan 24/26 mg BID  Tamsulosin 0.4 mg daily | STABLE/ FULL  84-y/o M with a PMH of DMT2, COPD, afib on AC, HFrEF (EF of 35%) s/p ICD placement, and gastroparesis admitted for concern for a gout flair in te right knee  Today:  -pain improved, cont on prednisone  -some confusion this afternoon, likely delirium  XC: NTD  If/then:  -if goes into RVR give 5 mg of metop |
