# Supplementary material for: Post In-Hospital Cardiac Arrest Simulation to Improve Medical Decision Making and Communication in Internal Medicine Residents
Source: MedEdPORTAL. 2026 Oct 16;22:11642. doi: 10.15766/mep_2374-8265.11642 (PMC13674318; doi:10.15766/mep_2374-8265.11642)
Supplement: Supplementary file 1 [file mep_2374-8265.11642-s001.zip › D. Case 1 Supplemental Data.docx]

**Appendix D: Case 1 Supplemental Data**

**Case 1 supplemental data** should be provided to simulation participants if requested. Laboratory diagnostic data should be cut apart prior to the simulation so that the specific laboratory studies requested by the participants can be provided. Diagnostic studies that are not requested by the participants should not be provided. All laboratory, electrocardiogram, and radiograph diagnostic data should be provided to the participants 1-2 minutes after being requested.

| **Point-of-Care Glucose** | 294 mg/dL |
| --- | --- |
| **Complete Blood Count** | WBC 23.94 K/uL, Hgb 12.3 g/dL, Hct 42%, Plts 512 K/uL |
| **Basic Metabolic Panel** | Na 124 mEq/dL, K 5.1 mEq/dL, Cl 92 mg/dL, HCO3 10 mEq/L, BUN 31 mg/dL, Cr 3.2 mg/dL, Ca 8.7 mg/dL, Mg 1.9 mg/dL |
| **B-type Natriuretic Peptide** | 35 pg/mL |
| **Troponin-I** | 41.8 ng/L |
| **Liver Function Tests** | AST 131 U/L, ALT 192 U/L, Alk Phos 132/ TBili 2.4 |
| **Coagulation Studies** | APTT 34.8 secs, PT 11.9 secs, INR 1.1 |
| **Arterial Blood Gas** | pH 7.12, pCO2 31 mmHg, pO2 127 mmHg , HCO3 11 mEq/L |
| **Lactate** | 6.3 mmol/L |

**Post Cardiac Arrest Chest X-Ray**

Image by Kirollos Bechay, retrieved from: https://radiopaedia.org/cases/pacer-pads-and-automated-implantable-cardioverter-defibrillator-in-intubated-patient?lang=us on 12/1/25. Licensed under https://creativecommons.org/licenses/by-nc-sa/3.0/.

**Chest radiograph from before hospitalization**

Image by Jeremy Jones, retrieved from: https://radiopaedia.org/cases/dual-lead-cardiac-pacemaker on 12/15/25. Licensed under https://creativecommons.org/licenses/by-nc-sa/3.0/.

**Post ROSC ECG, Stages 1 and 2**

**Case courtesy of life in the fast lane:** <https://litfl.com/atrial-fibrillation-ecg-library/> accessed on 12/15/25. Licensed under https://creativecommons.org/licenses/by-nc-sa/4.0/.

**Post ROSC ECG Stage 3**

**Provide this ECG to participants if the case has proceeded to stage 3.**

**Case courtesy of life in the fast lane:** <https://litfl.com/atrial-fibrillation-ecg-library/> accessed on 12/15/25. Licensed under https://creativecommons.org/licenses/by-nc-sa/4.0/.

**Admission ECG**

**Provide this electrocardiogram to participants if they request an electrocardiogram from the hospital admission.**

**Case courtesy of life in the fast lane:** <https://litfl.com/atrial-fibrillation-ecg-library/> accessed on 12/15/25. Licensed under https://creativecommons.org/licenses/by-nc-sa/4.0/.
