# Supplementary material for: Post In-Hospital Cardiac Arrest Simulation to Improve Medical Decision Making and Communication in Internal Medicine Residents
Source: MedEdPORTAL. 2026 Oct 16;22:11642. doi: 10.15766/mep_2374-8265.11642 (PMC13674318; doi:10.15766/mep_2374-8265.11642)
Supplement: Supplementary file 1 [file mep_2374-8265.11642-s001.zip › E. Debrief Guide.docx]

**Appendix E: Debrief Guide**

*This guide should be utilized to support the discussion of the debriefs that occur after each simulation. The debrief should focus on the applied experience of the participants during the simulation including their emotional response, communication and teamwork elements, and medical decision making. This should not be used as a time to review medical knowledge that was not directly related to the simulation experience. Discussions and questions about medical knowledge should otherwise be defered to the post ROSC framework section of the session.*

**Overall: 10-15 minutes**

1) **Frame the debrief:**
   - *Thank you to all our active participants! To debrief this simulation, we’ll focus on two aspects of leading an acutely decompensated hospitalized patient: The teamwork, and resource utilization, and then the medical knowledge and management side. To start out, are there any initial reactions to how that went?*
2) **Hand out Critical Action Checklists**
   - *This is a list of what we look for when our interns perform these simulations, This will give you a good idea of some of the specific team leadership and medical management actions you should perform with a patient with [PEA or VT post ROSC care]. We’ll review this as we talk about each part*
3) **Shared mental model**:
   - *Before we get into the debrief, Could someone summarize what you think was going on with the patient? It’s ok if you don't remember the specifics of the patient, I just want to see if we all on the same page about what was causing them to be altered.*
   - Attempt to obtain this description from someone other than the rapid response leader. If no responses, ask the rapid response leader
   - Following response with Who has another summary of the situation*?*
4) **Teamwork debrief –** this should be the larger portion of the debrief, the chalk talk will review some medical management
   - *We’ll start with the teamwork portion of the simulation.*
   - Some questions to start the discussion – can phrase questions to active participants and observers separately
     - *What do we think went well*
     - *What do we think did not go well?*
   - Points to hit/discuss
     - ***Announce yourself as the code blue team leader***
     - ***Ask for vital signs***
     - ***Assign roles***
     - ***Stand at foot of bed***
     - ***Closed loop communication***- also confirming task completion
     - ***Get help early –*** resident, attending, STAT nurse, MICU, anesthesia, RT etc
     - ***Share your thought process and ask the team for input early-*** create a **shared mental model**
     - *Avoid task saturating nursing staff* – asking for multiple tasks all at once; instead say “I will need A, B, and C. Could you start with A?”
     - *Ask for events leading up to the cardiac arrest*
     - *Asks for medication list, past medical history, recent labs*
5) **Communication debrief –**
   - participants should be asked to reflect on the communication that occurred between the active participants, to the standardized nurse, as well as to the standardized consultant.

Reflect on the quality of the sign-out that was given to the standardized consultant at the completion of the simulation using the SBAR framework as a guide.

**S = Situation** (what was the situation the participants found the patient in?)

**B = Background** (pertinent and brief information related to the situation)

**A = Assessment** (what are the critical elements what were discovered diagnostically, what is the pathology driving the patient presentation, what interventions were taken)

**R = Recommendation** (what are the next steps the participants would recommend)

6) **Medical Management debrief –** *What questions do you have about the medical treatment of this patient?*
   - **For learning points to highlight if not brought up by simulation participants, see each case below**
   - Importantly, the debrief should not simply re-teach content that was discussed during the chalk talk. The medical management debrief should include references to the content in the chalk talk in the context of the simulation case that provided challenges to the leaners, or corrective instructions on how the knowledge may have been incorrectly applied by the learners in the simulation.
   - For both cases, if the ACLS post-arrest care algorithm was not requested and reviewed during the simulation, discuss the use of cognitive aids such as this and where they can be found at your institution.

**Case 1: Post ROSC after Pulseless Electrical Activity Cardiac Arrest**

**Learning points/medical knowledge:**

- *PEA/ Asystole arrests are much more common for hospitalized patients. These cardiac arrests occur because the patient has a significant systemic pathology that overwhelms the patient's cardiac capacity.*
- *For PEA cardiac arrests for hospitalized patients, there should be a high suspicion for sepsis as the etiology.*
- *Patients presenting with distributive shock with concern for sepsis should be treated with IV crystalloid (30 mL/Kg), broad spectrum antibiotics, and possibly norepinephrine.*
- *Patients who have poor mental status responses, especially in the setting of hemodynamic instability, should be intubated for airway protection.*
- *Unstable tachyarrhythmias with a pulse (defined by hypotension, altered mentation, chest pain, or shortness of breath) should be managed with synchronized cardioversions.*

**Case 2: Post ROSC after Ventricular Tachycardia Cardiac Arrest**

**Learning points/medical knowledge:**

- *Patients with ventricular tachycardia cardiac arrests should be screened for cardiac ischemia as the cause of the cardiac arrest.*
- *These patients should get an ECG in the post ROSC setting, and cardiology should be consulted.*
- *Unstable tachyarrhythmias with a pulse (defined by hypotension, altered mentation, chest pain, or shortness of breath) should be managed with synchronized cardioversions.*
