# Supplementary material for: Post In-Hospital Cardiac Arrest Simulation to Improve Medical Decision Making and Communication in Internal Medicine Residents
Source: MedEdPORTAL. 2026 Oct 16;22:11642. doi: 10.15766/mep_2374-8265.11642 (PMC13674318; doi:10.15766/mep_2374-8265.11642)
Supplement: Supplementary file 1 [file mep_2374-8265.11642-s001.zip › F. Case 1 Critical Action Checklist.docx]

| **Appendix F: Case 1 Critical Action Checklist**  **Critical Action Checklist should be used by facilitators while observing the simulation and used to support the debrief discussion after the simulation is completed. The content of the checklist should not be shared with simulation participants before the simulation.** | | | | | | |
| --- | --- | --- | --- | --- | --- | --- |
| **Post ROSC Case 1 Critical Action Checklist** | | | | **Date:**  **Site:**  **Leader:**  **Others:** | | |
| **Evaluator:** | | | |  |  |  |
| **Code Blue Team Leadership** | | | |  |  |  |
| 1. Identifies self as leader of code blue team | | | | | |  |
| 2. Asks for updated vital signs | | | | | |  |
| 3. Stands at foot of bed | | | | | |  |
| 4. Assigns roles (or identifies roles) | | | | | |  |
| 5. Asks for events leading up to code team arrival | | | | | |  |
| 6. Asks for current medication list | | | | | |  |
| 7. Asks for past medical history | | | | | |  |
| 8. Asks for recent labs | | | | | |  |
| **Initial post ROSC assessment** | | | | | | |
| 1. Verbalizes differential diagnosis going through Hs and Ts | | | | | |  |
| 2. Obtains electrocardiogram | | | | | |  |
| 3. Obtains chest radiograph | | | | | |  |
| 4. Assesses neurological status (examines pupils, mental status, gross motor function) | | | | | |  |
| **Labs** | | | | | | |
| Basic metabolic panel |  | Complete blood count |  | | Blood gas with lactate |  |
| **Treatment/drugs** | | | | | | |
| 4. IV fluids | | | | | |  |
| 5. IV pressors such as norepinephrine | | | | | |  |
| 6. Broad spectrum antibiotics such as cefepime +metronidazole, piperacillin and tazobactam, or carbapenem +/- vancomycin | | | | | |  |
| **Other Comments:** | | | | | | |
