# Supplementary material for: Post In-Hospital Cardiac Arrest Simulation to Improve Medical Decision Making and Communication in Internal Medicine Residents
Source: MedEdPORTAL. 2026 Oct 16;22:11642. doi: 10.15766/mep_2374-8265.11642 (PMC13674318; doi:10.15766/mep_2374-8265.11642)
Supplement: Supplementary file 1 [file mep_2374-8265.11642-s001.zip › G. Post-ROSC Care Framework.docx]

Appendix G: Post-ROSC Care Framework

| **1: Primary Survey** | **2: Management** | **3.Communication**  **SBAR Hand off**  **Situation** (what happened?)  **Background** (what led to this decompensation?)  **Assessment** (diagnostics and therapeutics undertaken)  **Recommendation** (next steps)  **Inform primary team**  **Call DPOA**   - Medical update and provide empathic listening   **Debrief code team**   - What went well? What could be better? Any equipment failures?   **Check on neighboring patients**   - Acknowledge possible trauma,   **Document in EMR** |
| --- | --- | --- |
| **Exam**   - Vitals - Cardiovascular - Extremities (cold/warm) - Line/tube check   - Plan for Central venous catheter/Arterial-line - Mental status | **Optimize ventilator + oxygenation**   - SpO2 goal 90-98% - PaCO2 goal 35-45 mmHg |  |
|  | **Manage hemodynamics**   - MAP goal >65 mmHg - Fluids/ blood - Pressors/ inotropes - Treat arrhythmias |  |
| **Hs and Ts** Cause \| Study; Hypovolemia \| Exam; Hypoxia \| ABG; Acidosis (sepsis) \| ABG; Hyper/hypokalemia \| BMP; Tension pneumothorax \| Ultrasound; Tamponade \| Ultrasound; Thrombosis (Pulmonary embolism /coronary) \| ECG | **Other management**  No  No  Yes  Yes |  |

Myocardial infarction?

Coronary reperfusion

Start targeted temperature management

Patient following commands?

Ongoing stabilization
