# Supplementary material for: Post In-Hospital Cardiac Arrest Simulation to Improve Medical Decision Making and Communication in Internal Medicine Residents
Source: MedEdPORTAL. 2026 Oct 16;22:11642. doi: 10.15766/mep_2374-8265.11642 (PMC13674318; doi:10.15766/mep_2374-8265.11642)
Supplement: Supplementary file 1 [file mep_2374-8265.11642-s001.zip › H. Post-ROSC Care Framework Facilitator Guide.docx]

**Appendix H: Post-ROSC Framework Talk Facilitator Guide**

**Learning Objectives**

By the end of the chalk talk, learners will be able to:

1. Describe the initial primary survey and diagnostic evaluation of a patient who is post-ROSC
2. Differentiate between common causes of cardiac arrest
3. Name critical therapeutic and communication tasks in caring for a patient for the first 20 minutes of post-ROSC care

**1: Primary Survey**

**You are part of the code team and are caring for a patient who has just achieved return of spontaneous circulation after a cardiac arrest. What initial information do you want to know?**

Full set of vital signs

**What will you look for specifically when you examine the patient?**

- Cardiovascular exam
- Extremity exam (warm versus cool)
- Lines/tubes (evaluate for adequate venous access, arterial BP monitoring, airway needs, endotracheal tube)
- Mental status
- Note that patients will transfer to the ICU after code (if not already there)

*Have learners name specific organ systems/exam maneuvers to target in the exam. This focused exam should take ~1 minute.*

**What are common causes of cardiac arrest to consider for a hospitalized patient, and how would you rule them in or out for this patient?**

*Discuss that the differential for hospitalized patients is different from out of hospital cardiac arrest. For example hypothermia is an extremely unlikely cause of inhospital cardiac arrest, but acidosis due to septic shock is much more common.*

Review Hs and Ts, along with primary test or exam finding use to identify. Ideally, the learners should be sharing the Hs and Ts that they can recall to build the list. Highlight that after a cardiac arrest a BMP, blood base, CXR, ECG, and POCUS will be high yield diagnostic tests for all patients.

**Hs and Ts chart**

| **Cause** | **Study** | **Facilitator notes** |
| --- | --- | --- |
| Hypovolemia | Exam | Note that hemorrhagic losses need to be identified on exam not on labs in the acute setting |
| Hypoxia | Arterial blood gas | Note that pulse oximetry under SpO2 of 85% can be unreliable and that blood gas should be obtained |
| Acidosis (sepsis) | Arterial blood gas | Sepsis is a very common cause of PEA arrest in hospitals. Consider if an infectious work up is needed for all PEA arrest patients. |
| Hyper/hypokalemia | Basic metabolic panel | Correlate concerns for potassium causes with ECG findings |
| Tension pneumothorax | Ultrasound/Exam | Discuss the providers that should be called during the day and night to help manage this emergency |
| Tamponade | Ultrasound/Exam | Discuss the providers that should be called during the day and night to help manage this emergency |
| Thrombosis (Pulmonary embolism /coronary) | Electrocardiogram | Note that a PE can slip through your initial diagnostic work up |

**2: Initial management**

**Focus on respiratory system and hemodynamics**

- Airway management: Was the patient intubated? Do they need to be intubated?
- Goal oxygenation: SpO2 90-98%
- Goal ventilation: PaCO2 35-45 mmHg
- Maintain a MAP of 65 mmHg
  - Consider usual resuscitation with fluids/blood in addition to pressors/ ionotropes
  - Treat arrhythmias

**Other steps in management**

- *Stress that next steps in management can be complex and are best completed in consultation with critical care specialists*
- If MI, patient should go to cath lab for coronary intervention as soon as possible
- If patient is not following commands after ROSC, proceed with targeted temperature management (TTM) AFTER any coronary intervention.
  - Note that there is often variation about different patients receiving TTM at different institutions, but the AHA recommendations are for post ROSC comatose patients to be managed with TTM

**3: Communication**

**Who should be contacted after a code?**

- **Sign out to the accepting team in SBAR format**
  - **Situation** (what happened?)
  - **Background** (what led to this decompensation?)
  - **Assessment** (diagnostics and therapeutics undertaken during post ROSC care)
  - **Recommendation** (next steps: medications that need to be administered, procedures that need to take place, what needs to be ordered, who needs to be called)
- **Primary team**
  - Update the primary team on the situation
- **Family/DPOA**
  - This is a traumatic experience for the patient's family. Provide an update with plans for the next steps. Expect emotion and provide empathic listening.
- **Neighboring patients**
  - Patients who were in the same room as a patient who had a cardiac arrest can be traumatized by the experience. Check in with them to see how they are doing. Do not provide medical information about the patient. Validate their experience.
- **Debrief with the code team**
  - What went well? What could have been better? Where there any equipment or system challanges that we should be aware of?
- **Remember to document the EMR**
  - Include details about the cardiac arrest, post ROSC care, and what was communicated

**Take home points**

1. Initial vital signs and a focused exam provide valuable information quickly
2. Use ECG, ultrasound, labs, and exam to evaluate for common causes of cardiac arrest
3. Initial management should focus on optimizing ventilator settings and supporting hemodynamics
4. Communicate with other teams, family, and document in code note
5. Share the post ROSC cognitive aids are available through the AHA and are often placed on code carts for easy accessibility. <https://cpr.heart.org/-/media/cpr-files/cpr-guidelines-files/algorithms/algorithmacls_pcac_200622.pdf?la=en>
