# Supplementary material for: Post In-Hospital Cardiac Arrest Simulation to Improve Medical Decision Making and Communication in Internal Medicine Residents
Source: MedEdPORTAL. 2026 Oct 16;22:11642. doi: 10.15766/mep_2374-8265.11642 (PMC13674318; doi:10.15766/mep_2374-8265.11642)
Supplement: Supplementary file 1 [file mep_2374-8265.11642-s001.zip › I. Case 2 Facilitator Guide.docx]

**Appendix I: Case 2 Facilitator Guide**

| **Simulation case title: Return of Spontaneous Arrhythmias** | |
| --- | --- |
| **Patient name: Jennifer Gold**  **Patient Age: 79** | |
| **Brief narrative description of case** | Jennifer Gold is an 79-year-old woman with a past medical history of chronic kidney disease stage 3, atrial fibrillation, hypertension, hyperlipidemia, heart failure with reduced ejection fraction (EF of 15%), and current tobacco use admitted for a heart failure exacerbation. The patient had sustained ventricular tachycardia on telemetry and was pulseless when the bedside RN came to check on her. Before the learners entered the room, the patient was defibrillated and received one round of CPR before return of spontaneous circulation was obtained. |
| **Learning Objectives** | - Obtain and give hand off for a post ROSC patient to/from another medical team including relevant events leading up to the cardiac arrest, diagnostic results, therapeutics initiated, and differential diagnosis for the cardiac arrest. - Utilize a post ROSC checklist to support clinical decision making for a patient in the immediate post ROSC period. - Demonstrate the importance of obtaining a broad diagnostic work up in the immediate post ROSC period to determine any reversible causes of a cardiac arrest. - Choose initial therapeutics needed to stabilize a patient’s hemodynamics who has just obtained the return of spontaneous circulation as determined by a critical action checklist. |
| **Critical Actions** | - Identify self upon entering the room and assign roles. - Obtain sign out upon entering the room from medical team members. - Perform focused physical exam. - Obtain a broad diagnostic work up to determine the etiology of cardiac arrest that includes an ECG. - Articulate a differential diagnosis for the cardiac arrest that includes cardiac ischemia. - Treat ventricular tachycardia with amiodarone or direct cardioversion. - Call a code STEMI. - Call anesthesia for consideration for intubation. - Provide sign out the cardiology fellow. |
| **Page to rapid response leader** | “Code Blue, pt Gold” |
| **Patient Medications** | Acetaminophen 650 mg PO Q6H  Apixaban 5mg PO BID  Atorvastatin 80 mg PO daily  Furosemide 80 mg IV BID  Metoprolol Succinate 75 mg PO daily  Nicotine patch 21 mg daily  Sacubitril / Valsartan 24/26 mg PO BID  Senna PO PRN  Melatonin 5 mg QHS PO PRN |
| **Patient AM Labs** | WBC 4.6 K/uL, Hgb 12.8 g/dL, Hct 37%, Plts 214 K/uL    Na 134 mEq/L, K 3.6 mEq/L, Cl 95 mEq/L,  HCO3 15 mEq/L, BUN 32 mg/dL, Cr 2.9 mg/dL, Mg 1.8 mEq/L, Glucose 125 mg/dL |
| **Learner Preparation** | None |
| **Manikin setup on learner arrival** | - Monitor: patient is attached to monitor with pulse, blood pressure, oxygenation, and temperature displayed. - The patient is in bed at 0 degrees. - A chest compression backboard should be under the patient and defibrator pads are on the patient. - Blood pressure cuff is applied to patient. - Pulse oximeter is attached to patient - Pulse: patient has a regular tachycardic pulse with rate in ~130s - Access: one 20g PIV present in right AC - Exam: eyes are half open - Standardized STAT RN is feeling the left carotid pulse when learners enter. |
| **Standardized Participants** | - STAT RN: Gives sign out to learners and provides verbal cues to learners if needed - Consultant: both Cardiology fellow and anesthesia on the phone, presents to bedside at the end of the simulation |
| **Materials Needed** | - Manikin - Blood pressure cuff - IV Fluids - Medications including IV amiodarone 300 mg - Vasopressors - Defibrillator with pads - Copy of ACLS post arrest care algorithm |
| **Timing of medication arrival.** | • IV Fluids should be provided to the medical team immediately upon request.  • Medications should be provided 1-2 minutes after request. |
| **Timing of new data.** | • Laboratory data should be provided to the medical team 1 minute after requested.  • ECG should be provided at the time it is requested.  • CXR should be provided 1 minute after requested.  • Any requested culture data does not result during simulation.   - Ultrasound is out of service and cannot be utilized during this simulation. |

| **Physical Examination** | |
| --- | --- |
| **INITIAL VITAL SIGNS** | Temperate 37.6 C; Heart Rate 124 BP 90s/60s; MAP<65; RR 16; SpO2 93% on 4 liters of Oxygen/minute nasal canula |
| **General** | Eyes half open, arousable but confused to tactile or verbal stimulation |
| **Lungs** | Normal respiratory rate, no rhonchi, no wheezes, no cough |
| **Cardiovascular** | Tachycardic, regular rhythm, +S3 extra heart sound |
| **Abdomen** | Flat, non-distended, normal bowel sounds |
| **Neurological** | Arousable to tactile or verbal stimulation, AOx2 (name and place), no focal deficits on exam, pupils react to light appropriately |
| **Extremities** | 2+ edema to bilateral knees, extremities are warm to the touch |
| **Skin** | Warm, no rashes on skin |
| **Psychiatric** | Tangential, appropriate content, normal mood |

| **Case Stage/ Time Point** | **Case Details** | **Additional Information** |
| --- | --- | --- |
| **State 1: Learner enters room and begins care of patient.** | - T 36.2, HR 120s (sinus tachycardia), BP 90s/60s, MAP>65, RR 16, SpO2 93% on 4L NC - When learners enter the room STAT RN reports “the patient just had a VT arrest, we shocked her got ROSC, we did one round of CPR.” - The patient has a pulse throughout state 1. - Patient is confused but responsive to verbal cues. | **Trigger Points:**   - After learners receive ECG to interpret proceed to stage 2. |
| **State 2: Ventricular tachycardia** | - T 37.6, HR 140s ventricular tachycardia, BP 90s/50s, RR 18, SpO2 94% on 4L NC - The patient is in stable ventricular tachycardia; they have a pulse and can converse with the medical team though they are confused. - Rhythm does not change regardless of medical interventions administered, including 300 mg of IV amiodarone. | **Trigger Points:**   - Proceed to stage 3 after 8 minutes. |
| **State 3: Patient with deteriorating hemodynamics.** | - Vital signs: BP 60s/40s, HR 130-140s ventricular tachycardia, O2 saturation 60s% on 4L NC. - The patient has decreasing responsiveness to verbal or tactile stimulation. - The patient has a pulse throughout state 3. - If the patients perform a synchronized cardioversion that patient will return to sinus tachycardia with a heart rate in the 110s. Blood pressure improves to 90s/60s. | **Trigger Points:**   - After 10 minutes proceed to state 4   **OR proceed to state 4 earlier if perform the following actions:**   - Synchronized cardioversion of the patient. - Call cardiology for a code STEMI |
| **State 4: Hand off to the cardiology fellow** | - A standardized cardiology fellow enters the room and asks for a summary of the situation. - If not discussed, cardiology fellow should ask learners for events of the cardiac arrest, diagnostics obtained, therapies started, and differential diagnosis. |  |
| **Case Ends** |  |  |

**Ideal Scenario Flow***.*

The learners enter the room to find a patient who has just had a ventricular tachycardia cardiac arrest and recieved one shock with a defibrillator and one round of CPR prior to return of spontaneous circulation being achieved. The learners obtain an electrocardiogram, and they verbalize a concern for ST segment elevations. Immediately after obtaining the ECG, the patient’s cardiac rhythm goes from sinus tachycardia to stable ventricular tachycardia. The learners bolus the patient with 300 mg of IV amiodarone but there is no change in the patient's hemodynamics. The patient’s blood pressure continues to decline, and their mental status deteriorates. The learners recognize that the patient is in an unstable ventricular tachycardia and perform a synchronized cardioversion. After the cardioversion the patient's hemodynamics improve. The learners consult cardiology and when the standardized cardiologist arrives the learners sign out their concern for cardiac ischemia.

**Anticipated Management Mistakes**

1. Learners perform unsynchronized cardioversion while patient has a pulse: Learners have been confused about the indications for synchronized vs unsynchronized cardioversion. Despite the patient having a pulse in this case, learners have performed an unsynchronized cardioversion. We have had the patient go into ventricular fibrillation without a pulse when this occurs.
2. Delayed diagnostics. If participants do not ask for diagnostics tests, including an ECG within 4 minutes of the simulation starting, they can be promoted to do so by the standardized STAT RN.
3. Failure to consult the necessary teams. Some simulation participants do not consult cardiology. If this occurs, the standardized STAT RN can prompt the participants to consult these teams verbally suggesting it after 7 minutes within the simulation.
4. Medical management of an unstable tachyarrhythmia. Learners have attempted to medically manage with patient in state 3 where the patient is presenting with an unstable tachyarrhythmia. The patient's hemodynamics continues to deteriorate if this form of management is attempted. If the learners want to administer a second medication after the first has not worked, the standardized STAT RN can suggest that the patient should be cardioverted.

**Cardiology fellow consult:** If the learners call for a cardiology consult, they should be provided a phone number to be able to reach the standardized cariology fellow who should be in a different room than the simulation. Upon picking up the phone the standardized cariology fellow should obtain information regarding key patient past medical history, events leading up to the cardiac arrest, details of the cardiac arrest, as well as diagnostic data obtained after ROSC was achieved. These details should be requested if they are not offered by the learner making the call. Learners should be asked to provide their interpretation of the patient's ECGs since ROSC was obtained. After this information is obtained, the standardized cardiology fellow should tell the learner they will be at bedside shortly and that the cardiac catheterization lab is being activated.

**Anesthesia consultant:** If the learners call for anesthesia, they should be provided with a phone number to be able to reach anesthesia who should be in a different room than the simulation. Upon picking up the phone the standardized anesthesiologist should obtain information regarding key patient past medical history, events leading up to the cardiac arrest, details of the cardiac arrest, as well as diagnostic data obtained after ROSC was achieved. Learners should provide their thoughts on whether intubation is required now and the reasons why. After this information is obtained, the standardized anesthesiologist should tell the learner they will be at bedside shortly.

**Case 2 supplemental data** should be provided to simulation participants if requested. Laboratory diagnostic data should be cut apart prior to the simulation so that the specific laboratory studies requested by the participants can be provided. Diagnostic studies that are not requested by the participants should not be provided. All laboratory, electrocardiogram, and radiograph diagnostic data should be provided to the participants 1-2 minutes after being requested.

| **Point-of-Care Glucose** | 193 mg/dL |
| --- | --- |
| **Complete Blood Count** | WBC 7.94 K/uL, Hgb 11.3 12.3 g/dL, Hct 36% Plts 212 K/uL |
| **Basic Metabolic Panel** | Na 129 mEq/dL, K 3.7 mEq/dL, Cl 92 mg/dL, HCO3 19 mEq/L, BUN 45 mg/dL, Cr 3.2 mg/dL, Ca 8.7mg/dL, Mg 1.6 mg/dL |
| **B-type Natriuretic Peptide** | 159 pg/mL |
| **Troponin-I** | 177.143 ng/L |
| **Liver Function Tests** | AST 131 U/L, ALT 192 U/L U/L, AlkPhos 132, TBili 2.4 |
| **Coagulation Studies** | APTT 31.8 secs, PT 12.1 secs, INR 1.4 |
| **Arterial Blood Gas** | PH7.26/ pCO2 31 mmHg / pO2 63mmHg/20 11 mEq/L |
| **Lactate** | 2.9 mmol/L |

**X Ray from before hospitalization**

Image courtesy of Sarmad Aslam, Radiopaedia.org, rID: 79296 https://radiopaedia.org/cases/cardiomegaly-8?lang=us, accessed on 12/15/25. Licensed under https://creativecommons.org/licenses/by-nc-sa/3.0/.

**Post Cardiac Arrest Chest X-Ray**

Image courtesy of Kirollos Bechay, Radiopaedia.org, rID: 151245, https://radiopaedia.org/cases/pacer-pads-and-automated-implantable-cardioverter-defibrillator-in-intubated-patient?lang=us accessed on 12/15/25. Licensed under https://creativecommons.org/licenses/by-nc-sa/3.0/.

**Admission ECG**

Case courtesy of life in the fast lane: https://litfl.com/atrial-fibrillation-ecg-library/ accessed on 12/15/25. Licensed under https://creativecommons.org/licenses/by-nc-sa/4.0/.

**Post ROSC ECG, Stage 1**

Case courtesy of life in the fast lane: https://litfl.com/lateral-stemi-ecg-library/. Accessed on 12/15/25. Licensed under https://creativecommons.org/licenses/by-nc-sa/4.0/.

**Post ROSC ECG, Stage 2 and 3**

Case courtesy of life in the fast lane: https://litfl.com/ventricular-tachycardia-monomorphic-ecg-library/ accessed on 12/15/25. Licensed under https://creativecommons.org/licenses/by-nc-sa/4.0/.

**Case 2 patient sign out data.** This document should be provided to participants at the beginning of the simulation.

| **FLOOR – 2W** | **VITALS-LABS** | **Ins/Outs – Cultures** | **MEDS** | **COMMENTS/TASKS** |
| --- | --- | --- | --- | --- |
| Gold, Jennifer  MRN01010101  DOB: 9/6/45, 79 F  6212-1  **Code: Full Code**  **Allergy: NKDA**  **Hospital Day 2**   1- Osteomyelitis   Prob/Proc   - CKD 3 - HTN - Atrial Fibrillation - HLD | T 37.8 p 98 NIBP 95/64 r18 sat 99% on RA weight 214 lb  WBC 5.3 K/uL, Hgb 13g/dL, Hct 42%, Plts315 K/uL    Na 134 mEq/L, K 3.9 mEq/L, Cl 97 mEq/L,  HCO3 15 mEq/L, BUN 32 mg/dL, Cr 2.9 mg/dL, Mg 1.8 mEq/L  Glucose 155 mg/dL |  | Acetaminophen 650 mg PO Q6H  Apixaban 5mg PO BID  Atorvastatin 80 mg PO daily  Furosemide 80 mg IV BID  Metoprolol Succinate 75 mg PO daily  Nicotine patch 21 mg daily  Sacubitril / Valsartan 24/26 mg PO BID  Senna PO PRN  Melatonin 5 mgQHS PO PRN | STABLE/ FULL  79-y/o W w/ PMH of CKD stage 3, atrial fib, HTN, HLD, HFrEF (EF of 15%), and current tobacco here with HF exacerbation  Today:  -cont IV Furosemide  80 BID  -down 24 lbs since admission  XC: NTD  OK to leave floor to smoke  If/then:  -if goes into RVR give 5 mg of metop |

| **Critical Action Checklist should be used by facilitators while observingthe simulation and used to support the debrief discussion after the simulation is completed. The content of the checklist should not be shared with simulation participants before the simulation.** | | | | | | | | |
| --- | --- | --- | --- | --- | --- | --- | --- | --- |
| **Post ROSC Case 2 Critical Action Checklist** | | | | | **Date:**  **Site:**  **Leader:**  **Others:** | | | |
| **Evaluator:** | | | | |  |  |  |  |
| **Code Team Leadership** | | | | |  |  |  |  |
| 1. Identifies self as leader of code team | | | | | | | |  |
| 2. Asks for vital signs | | | | | | | |  |
| 3. Stands at foot of bed | | | | | | | |  |
| 4. Assigns roles (or identifies roles) | | | | | | | |  |
| 5. Asks for events leading up to cardiac arrest | | | | | | | |  |
| 6. Asks for current medication list | | | | | | | |  |
| 7. Asks for past medical history | | | | | | | |  |
| 8. Asks for recent labs | | | | | | | |  |
| **Initial Post ROSC Assessment** | | | | | | | | |
| 1.  Verbalizes differential diagnosis including cardiac ischemia | | | | | | | |  |
| 2.  Obtains electrocardiogram | | | | | | | |  |
| 3. Obtains chest radiograph | | | | | | | |  |
| 4. Assesses neurological status (examines pupils, mental status, gross motor function) | | | | | | | |  |
| **Labs** | | | | | | | | |
| Basic metabolic panel |  | Complete blood count |  | Blood gas with lactate | |  | Troponin |  |
| Treatment/drugs | | | | | | | | |
| 1. IV fluids | | | | | | | |  |
| 2. Amiodarone | | | | | | | |  |
| 3. Synchronized Cardioversion | | | | | | | |  |
| **Other Comments:** | | | | | | | | |
