# Supplementary material for: Post In-Hospital Cardiac Arrest Simulation to Improve Medical Decision Making and Communication in Internal Medicine Residents
Source: MedEdPORTAL. 2026 Oct 16;22:11642. doi: 10.15766/mep_2374-8265.11642 (PMC13674318; doi:10.15766/mep_2374-8265.11642)
Supplement: Supplementary file 1 [file mep_2374-8265.11642-s001.zip › J. Case 2 Patient Sign Out.docx]

**Appendix J: Case 2 Patient Sign Out Data**

This document should be provided to participants at the beginning of the simulation.

| **FLOOR – 2W** | **VITALS-LABS** | **Ins/Outs – Cultures** | **MEDS** | **COMMENTS/TASKS** |
| --- | --- | --- | --- | --- |
| Gold, Jennifer  MRN01010101  DOB: 9/6/45, 79 F  6212-1  **Code: Full Code**  **Allergy: NKDA**  **Hospital Day 2**   1- Osteomylsis   Prob/Proc   - CKD 3 - HTN - Atrial Fibrillation - HLD | T 37.8 p 98 NIBP 95/64 r18 sat 99% on RA weight 214 lb  WBC 5.3 K/uL, Hgb 13 g/dL, Hct 42%, Plts 315 K/uL    Na 134 mEq/L, K 3.9 mEq/L, Cl 97 mEq/L,  HCO3 15 mEq/L, BUN 32 mg/dL, Cr 2.9 mg/dL, Mg 1.8 mEq/L  Glucose 155 mg/dL |  | Acetaminophen 650 mg PO Q6H  Apixaban 5mg PO BID  Atorvastatin 80 mg PO daily  Furosemide 80 mg IV BID  Metoprolol Succinate 75 mg PO daily  Nicotine patch 21 mg daily  Sacubitril / Valsartan 24/26 mg PO BID  Senna PO PRN  Melatonin 5 mg QHS PO PRN | STABLE/ FULL  79-y/o W w/ PMH of CKD stage 3, atrial fib, HTN, HLD, HFrEF (EF of 15%), and current tobacco here with HF exacerbation  Today:  -cont IV Furosemide  80 BID  -down 24 lbs since admission  XC: NTD  OK to leave floor to smoke  If/then:  -if goes into RVR give 5 mg of metop |
