# Supplementary material for: Post In-Hospital Cardiac Arrest Simulation to Improve Medical Decision Making and Communication in Internal Medicine Residents
Source: MedEdPORTAL. 2026 Oct 16;22:11642. doi: 10.15766/mep_2374-8265.11642 (PMC13674318; doi:10.15766/mep_2374-8265.11642)
Supplement: Supplementary file 1 [file mep_2374-8265.11642-s001.zip › K. Case 2 Supplemental Data.docx]

**Appendix K: Case 2 Supplemental Data**

**Case 2 supplemental data** should be provided to simulation participants if requested. Laboratory diagnostic data should be cut apart prior to the simulation so that the specific laboratory studies requested by the participants can be provided. Diagnostic studies that are not requested by the participants should not be provided. All laboratory, electrocardiogram, and radiograph diagnostic data should be provided to the participants 1-2 minutes after being requested.

| **Point-of-Care Glucose** | 193 mg/dL |
| --- | --- |
| **Complete Blood Count** | WBC 7.94 K/uL, Hgb 11.3 12.3 g/dL, Hct 36% Plts 212 K/uL |
| **Basic Metabolic Panel** | Na 129 mEq/dL, K 3.7 mEq/dL, Cl 92 mg/dL, HCO3 19 mEq/L, BUN 45 mg/dL, Cr 3.2 mg/dL, Ca 8.7 mg/dL, Mg 1.6 mg/dL |
| **B-type Natriuretic Peptide** | 159 pg/mL |
| **Troponin-I** | 177.143 ng/L |
| **Liver Function Tests** | AST 131 U/L, ALT 192 U/L U/L, Alk Phos 132, TBili 2.4 |
| **Coagulation Studies** | APTT 31.8 secs, PT 12.1 secs, INR 1.4 |
| **Arterial Blood Gas** | PH7.26/ pCO2 31 mmHg / pO2 63 mmHg/20 11 mEq/L |
| **Lactate** | 2.9 mmol/L |

**X Ray from before hospitalization**

Image courtesy of Sarmad Aslam, Radiopaedia.org, rID: 79296 https://radiopaedia.org/cases/cardiomegaly-8?lang=us, accessed on 12/15/25. Licensed under https://creativecommons.org/licenses/by-nc-sa/3.0/.

**Post Cardiac Arrest Chest X-Ray**

Image courtesy of Kirollos Bechay, Radiopaedia.org, rID: 151245, https://radiopaedia.org/cases/pacer-pads-and-automated-implantable-cardioverter-defibrillator-in-intubated-patient?lang=us accessed on 12/15/25. Licensed under https://creativecommons.org/licenses/by-nc-sa/3.0/.

**Admission ECG**

Case courtesy of life in the fast lane: https://litfl.com/atrial-fibrillation-ecg-library/ accessed on 12/15/25. Licensed under https://creativecommons.org/licenses/by-nc-sa/4.0/.

**Post ROSC ECG, Stage 1**

Case courtesy of life in the fast lane: https://litfl.com/lateral-stemi-ecg-library/. Accessed on 12/15/25. Licensed under https://creativecommons.org/licenses/by-nc-sa/4.0/.

**Post ROSC ECG, Stage 2 and 3**

Case courtesy of life in the fast lane: https://litfl.com/ventricular-tachycardia-monomorphic-ecg-library/ accessed on 12/15/25. Licensed under https://creativecommons.org/licenses/by-nc-sa/4.0/.
