# Supplementary material for: Post In-Hospital Cardiac Arrest Simulation to Improve Medical Decision Making and Communication in Internal Medicine Residents
Source: MedEdPORTAL. 2026 Oct 16;22:11642. doi: 10.15766/mep_2374-8265.11642 (PMC13674318; doi:10.15766/mep_2374-8265.11642)
Supplement: Supplementary file 1 [file mep_2374-8265.11642-s001.zip › M. Pre- and Postsimulation Survey.docx]

**Appendix M: Pre- and Post-Simulation Questionnaire**

| **Rate your level of confidence with the following:** | | | | | | | | |
| --- | --- | --- | --- | --- | --- | --- | --- | --- |
| Managing a post-ROSC patient after a PEA and/or asystolic cardiac arrest | Not at all | Not very | | Neutral | | Somewhat | | Very |
| Managing a post-ROSC patient after a ventricular tachycardia and/or ventricular fibrillation cardiac arrest | Not at all | Not very | | Neutral | | Somewhat | | Very |
| Providing handoff to a medical provider on a patient who has just obtained ROSC | Not at all | Not very | | Neutral | | Somewhat | | Very |
| Using a defibrillator | Not at all | Not very | | Neutral | | Somewhat | | Very |
| Generating a differential diagnosis for a patient’s cardiac arrest | Not at all | Not very | | Neutral | | Somewhat | | Very |
| **Please answer the following** | | | | | | | | |
| What is the recommended pCO2 for a patient in the post-ROSC setting? | 25-35 mmHg | | 35-45 mmHg**^a^ | | 45-50 mmHg | | 50-60 mmHg | |
| What is the recommended MAP for a patient in the post-ROSC setting? | >55 | | >60 | | >65**^a^ | | >70 | |
| What is the recommended SpO2 for a patient in the post-ROSC setting? | 88-92% | | >92% | | 94-97%**^a^ | | >98% | |

^a^** Indicates correct answer
